# Behavioural responses of humpback whales to food-related chemical stimuli

**DOI:** 10.1371/journal.pone.0212515

**Published:** 2019-02-26

**Authors:** Bertrand Bouchard, Jean-Yves Barnagaud, Marion Poupard, Hervé Glotin, Pauline Gauffier, Sara Torres Ortiz, Thomas J. Lisney, Sylvie Campagna, Marianne Rasmussen, Aurélie Célérier

**Affiliations:** 1 Behavioural Ecology Group, CEFE UMR 5175, CNRS–Université de Montpellier–Université Paul-Valéry Montpellier–EPHE, Montpellier, France; 2 Université de Montpellier, Montpellier, France; 3 DYNI team, LIS, Université de Toulon, Université Aix-Marseille, CNRS, Marseille, France; 4 CIRCE, Conservation, Information and Research on Cetaceans, Algeciras-Pelayo, Cadiz, Spain; 5 Marine Biological Research Centre, Department of Biology, University of Southern Denmark, Kerteminde, Denmark; 6 Université de Nîmes, Nîmes, France; 7 Húsavík Research Centre, University of Iceland, Húsavík, Iceland; University of Windsor, CANADA

## Abstract

Baleen whales face the challenge of finding patchily distributed food in the open ocean. Their relatively well-developed olfactory structures suggest that they could identify the specific odours given off by planktonic prey such as krill aggregations. Like other marine predators, they may also detect dimethyl sulfide (DMS), a chemical released in areas of high marine productivity. However, dedicated behavioural studies still have to be conducted in baleen whales in order to confirm the involvement of chemoreception in their feeding ecology. We implemented 56 behavioural response experiments in humpback whales using two food-related chemical stimuli, krill extract and DMS, as well as their respective controls (orange clay and vegetable oil) in their breeding (Madagascar) and feeding grounds (Iceland and Antarctic Peninsula). The whales approached the stimulus area and stayed longer in the trial zone during krill extract trials compared to control trials, suggesting that they were attracted to the chemical source and spent time exploring its surroundings, probably in search of prey. This response was observed in Iceland, and to a lesser extend in Madagascar, but not in Antarctica. Surface behaviours indicative of sensory exploration, such as diving under the stimulus area and stopping navigation, were also observed more often during krill extract trials than during control trials. Exposure to DMS did not elicit such exploration behaviours in any of the study areas. However, acoustic analyses suggest that DMS and krill extract both modified the whales’ acoustic activity in Madagascar. Altogether, these results provide the first behavioural evidence that baleen whales actually perceive prey-derived chemical cues over distances of several hundred metres. Chemoreception, especially olfaction, could thus be used for locating prey aggregations and for navigation at sea, as it has been shown in other marine predators including seabirds.

## Introduction

For filter-feeding animals such as baleen whales (mysticetes), finding patchily-distributed krill aggregations is a challenging task that involves movements over hundreds to thousands of kilometres of open ocean. The cues that they use to find food are still unclear, but baleen whales are thought to rely on multimodal signals when foraging, possibly using chemoreception in addition to acoustic and visual cues [1]. Chemical senses, and especially olfaction, play a key role in the foraging ecology of several marine predators feeding on similar planktonic prey [2]. For example, procellariform birds including Cape Petrel (*Daption capense*) and filter-feeding whale sharks (*Rhincodon typus*) detect prey-derived chemicals such as krill extracts in air and in water, respectively [3,4]. These species are also attracted by dimethyl sulfide (DMS), a molecule emitted in significant quantities by a range of phytoplankton taxa (primarily dinoflagellates and Prymnesiophyceae) when grazed by zooplankton [5–7]. DMS is now recognized as an efficient indicator of high marine productivity that play a crucial role in marine trophic interactions [8].

Anatomical studies have revealed that in contrast to toothed whales (odontocetes), baleen whales appear to have a functional olfactory system [9]. Unlike odontocetes, mysticete skulls possess a nasal cavity that contains well-developed ethmoturbinates (scroll-shaped bony protrusions that are covered with nasal mucosa) plus a chamber that houses the olfactory bulbs [10–12]. Although there is some discrepancy in the historical literature regarding the presence of olfactory bulbs among species, it is highly likely that these structures are present in all mysticetes, but are often lost during dissection [10]. Indeed, recent work on the bowhead whale (*Balaena mysticetus*) has confirmed the presence of large, well-developed olfactory bulbs in this species [13]. Genetic studies have also revealed that mysticetes have a high proportion of functional genes coding for olfactory receptors (OR), which are transmembrane proteins responsible for odorant binding expressed at the surface of olfactory neurons [13–15]. Mysticetes are thus thought to have a functional sense of smell, but this hypothesis has yet to be tested experimentally. A preliminary study [16] conducted on humpback whales (*Megaptera novaeangliae*), a species that feeds on krill as well as several schooling fish species depending on prey availability [17,18], showed that feeding humpback whales oriented into the wind significantly more often than in other directions. This suggests that foraging mysticetes could follow wind-borne molecules emitted by their prey, even if other chemosensory systems could also be involved, such as the perception of soluble compounds in water through gustation [1,19]. However, rigorous behavioural experiments are still necessary to confirm that whales can perceive chemical compounds and in particular food-related cues. Because mysticetes are not held in captivity, these experiments have to be conducted under the constraints imposed by the natural environment, while allowing a detailed measurement of the whales’ reactions towards potential chemical cues at sea (e.g. changes in their swimming trajectories, surface behaviours and acoustic activity).

In this study, we investigated the reactions of humpback whales to two food-related chemicals: krill extract and DMS. We tested whether these animals would be attracted by these stimuli, as indicated by a change in their swimming trajectories or speed, an increase in their respiratory rate (to improve airborne molecule sampling), and/or the display of specific feeding behaviours such as opening their mouths or making foraging dives. We also recorded the whales’ vocalizations since they are known to produce various sounds while foraging [20–22] and could thus display a specific acoustic response to such stimulation. We used two different chemical stimuli with different physical properties because mysticetes’ ability to perceive chemical stimuli may depend on the volatility/solubility of these compounds. Krill extract directly reflects the prey’s chemical signature and contains various volatile and soluble molecules. This stimulus could thus be perceived in air and/or in water depending on the anatomical location of chemoreceptors (e.g. in the nasal and/or oral cavity). In contrast, DMS is a highly volatile molecule transported over great distances by wind, and so is more likely to be detected in air during ventilation. DMS is considered as an indirect indicator of potential prey aggregation [5]. We therefore predicted that krill extract would be likely to have a higher attraction potential for humpback whales because it is a direct cue for the presence of prey. In this study, we focused on short term response (within less than half an hour of exposure) at a fine scale (hundreds of metres from the chemical source). This eliminated the potential issue of excessive dilution of the experimental chemical stimuli and thus allowed us to maximize the chances of observing behavioural reactions specific to these potential cues, even if it is clear that chemoreception may be used at much larger scales [1]. We conducted behavioural response experiments in three geographically distinct study areas that have different prey availabilities and which are related to different parts of the whales’ life cycles: one breeding ground (Madagascar, Indian Ocean) where the whales breed and calve and where limited food is available, as well as two feeding grounds (Iceland in the Northern Atlantic Ocean and the Antarctic Peninsula). We predicted that the whales would exhibit behavioural reactions towards food-related chemicals exclusively in their feeding grounds where they are actively searching for food, but not in their breeding grounds, where their behaviour is mostly focused on reproduction. A comparison of the whales’ responses to krill extract and DMS in these three areas should therefore explain for the first time how environmental and physiological factors influence the behavioural responses to food-related chemical stimuli in mysticetes.

## Materials and methods

### Study sites

**Fig 1:**
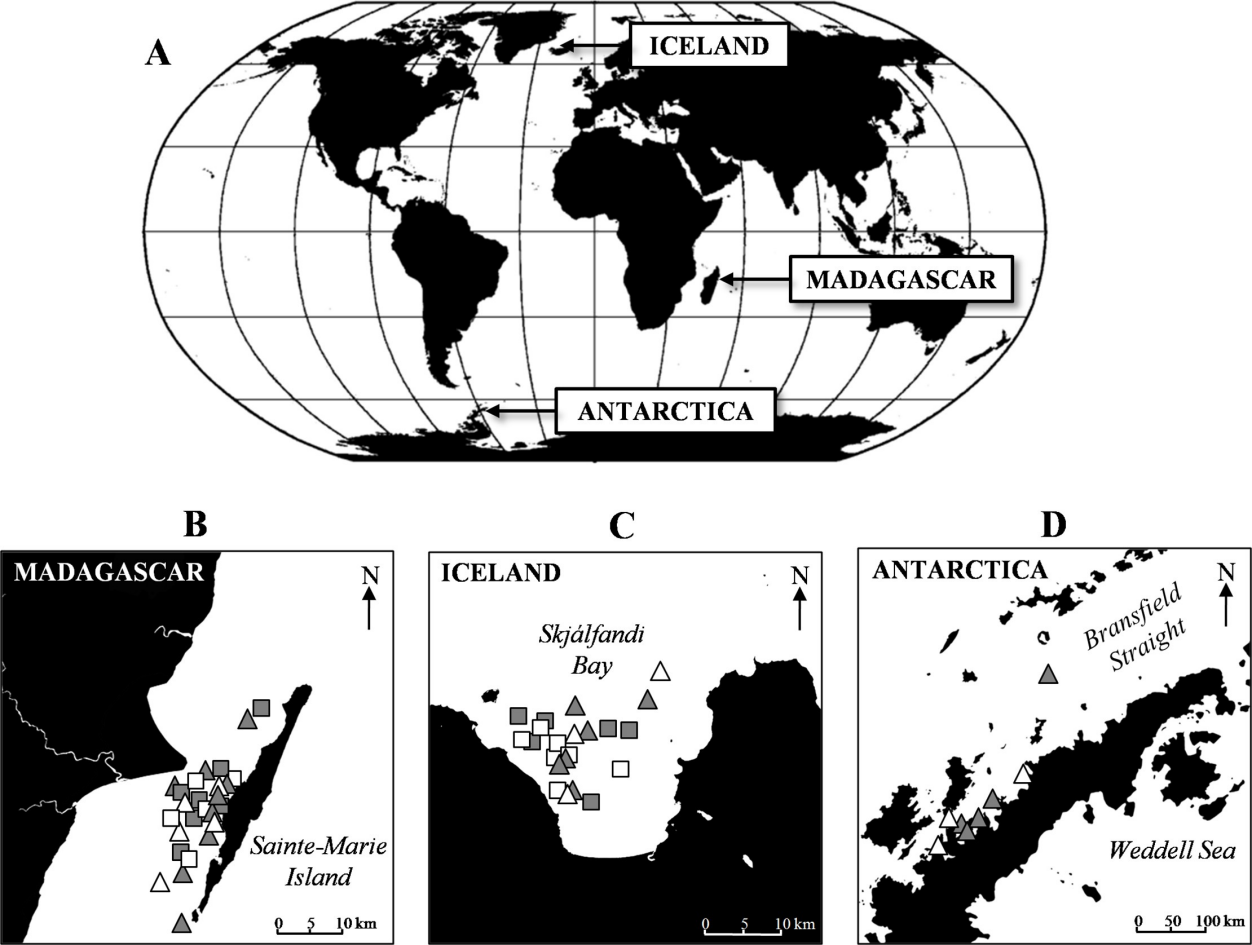
Maps of study areas and chemical stimulation sites. The three study areas are located on a world map (**A**). The location of the chemical stimulation trials are detailed for each area (**B**, **C** and **D**). Krill extract trials are represented by a triangle while DMS trials are represented by a square. Controls and chemical stimulations are shown in white and grey, respectively.

During 2015 and 2016, we carried out four fieldwork campaigns in the three different humpback whale breeding and feeding grounds (Fig 1A). For the breeding ground study area, we selected the Sainte Marie Channel (Fig 1B), located between Sainte Marie Island and the main island of Madagascar. The waters here are shallow and allow easy access to a high number of humpback whales at predictable times during the austral winter [23,24]. At this location, we implemented experiments from late June to early July in 2015 and 2016. For the feeding grounds study areas, we selected two different sites characterized by different kinds of prey availability. We carried out the first set of experiments in August 2016 on the north-eastern coast of Iceland in Skjálfandi Bay (Fig 1C), where humpback whales mostly feed on schools of small fish (herring and capelin) as well as krill [18,25]. We then conducted the second set of experiments in December 2016 around the Antarctic Peninsula (Fig 1D) where krill is the main food available for mysticetes [26,27]. This study was approved by the French national ethical committee (Comité Consultatif National d'Ethique, permit number: 1286.5392) and was in accordance with the European directive 86/609/CEE. Field work was conducted in accordance with permits issued by Direction Générale des Ressources Halieutiques et de la Pêche in Madagascar (permit numbers: 46/15 MRHP/DGRHP and 28/16 MRPH/DGRHP) and Administration Supérieure des Terres Australes et Antarctiques Françaises in Antarctica (permit number: arrêté 2016–139). No research permit was needed for the study of humpback whales in Iceland since the study was only observational, without invasive sampling method and since the species is not protected in the country.

### Targeted whale groups

In order to maximize the chances of finding whales at sea, detect their behaviours and get high quality observations, we carried out the experiments only under favourable weather conditions (i.e. no precipitation, visibility of more than 5 km, moderate winds (Beaufort’s wind scale force < 4) and swell less than 1.5 m). In all study areas, the research crew included two experienced cetacean observers, one bird observer and one experimenter. We adapted the protocol from a previous study on odontocete chemoreception [28]. Briefly, at the beginning of the day, the research boat navigated within the study area until a whale or a group of whales was opportunistically spotted. We then slowly approached the animals from the side and observed them from a distance of 300 m for 10 minutes. This distance is known to limit disturbance of natural behaviour in humpback whales [29]. This pre-trial observation phase allowed us to assess the number of whale groups in the area, with a group being defined as several individuals separated by less than 4 body lengths (about 50 m), generally moving in the same direction and showing coordination in their behaviour [30,31]. We took a whale group as a single sampling unit instead of an individual whale because previous reports and preliminary observations revealed that the movement and behaviour of individual whales within a group is highly dependent on that of the other group members [30]. Each group was assigned a unique code and we recognized individual groups during each trial on the basis of group size and specific physical features of the group members (body size, dorsal and caudal fin shape, coloration and markings). We also recorded the group size (number of individuals), their age category (calf, juvenile or adult) and initial behaviour. We defined three categories of initial behaviour: travelling (moving in one direction), resting (floating at the surface or moving very slowly) or diving (repeated dives with no significant progression towards a particular direction, sometimes surfacing with the mouth open suggesting a foraging activity) [32]. We made the decision to start a trial only if the whales’ behaviour was not obviously disturbed by the boat presence (e.g., no evidence of strong avoidance, agonistic surface behaviours such as caudal peduncle throws, or long dives) and if no other vessels (for example whale-watching boats or ferries) were likely to approach within a 2 km radius around the whales during the next 30 minutes.

### Chemical stimuli

Two chemical stimuli were tested: krill extract (experiment K) and DMS (experiment D). Because these two compounds exhibit different physical and chemical characteristics, two different exposure experiments were designed, each including both test and control trials.

#### Experiment K: Krill extract

For the krill extract experiments, we used 4 kg of a powdered hydrolysate of Antarctic krill (ground krill digested by subtilisin enzyme; Phosphotech, France) diluted in 8 litres of seawater as a chemical stimulus. As krill contains about 80% moisture [33], this dose approximately corresponds to the dry matter of 20 kg of fresh krill, which represents 2–5% of an adult humpback whale’s daily intake [34]. We made a control solution of similar colour (to control for the potential use of visual cues by the whales) using 60 g of an orange clay powder with no biologically relevant odour (Terracotta, Cultura, France) dissolved in the same amount of water.

**Fig 2:**
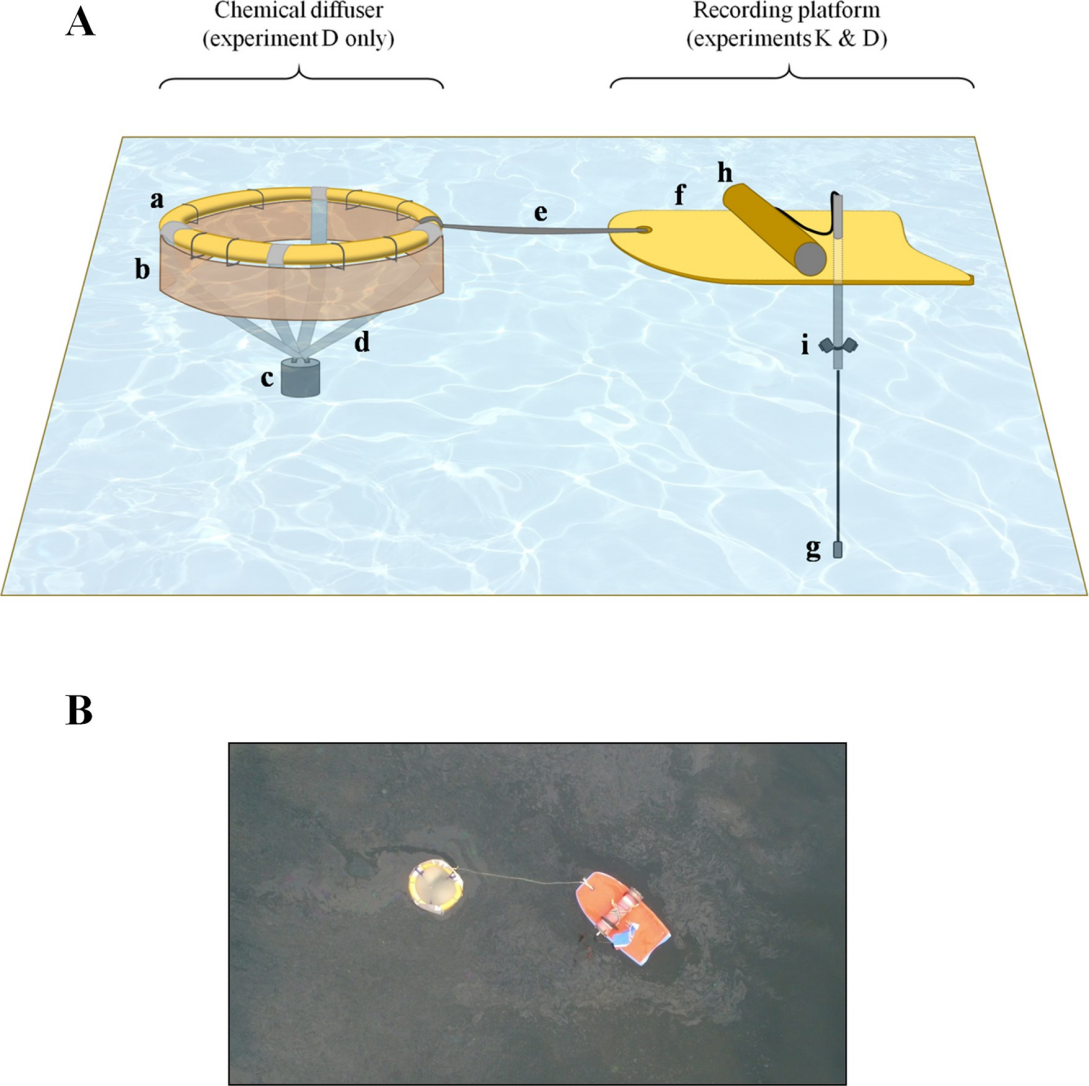
Details of the floating diffuser and recording platform used in the behavioural response experiments. (**A**) Schematic illustration of the chemical diffuser and the recording platform. The diffuser was used only during the DMS experiments and their controls (experiment D). It had a diameter of 80 cm and was made of one stainless steel-reinforced orange Styrofoam tube (**a**). A 30 cm long polyvinyl chloride membrane (**b**) was hung below the tubes in order to partly retain the oily solutions during the trial. A 1 kg lead weight (**c**) was hung 40 cm under the device by four polyester straps (**d**) and worked as an anchor in order to prevent excessive drifting due to wind. The diffuser was attached to the recording device, a 120 x 60 cm floating Styrofoam platform (**f**), by a 100 cm long strap (**e**). This platform was equipped with a hydrophone (model C57, Cetacean Research Technology, Seattle, USA) (**g**) connected to a digital audio recorder (Zoom H1 recorder, Zoom Corp., Tokyo, Japan) (**h**) and two underwater cameras (GoPro Hero 4, GoPro Inc., San Mateo, USA) (**i**) facing forward and backward with an angle of 30° downward. (**B)** Drone photograph of the floating diffuser and the recording platform during a DMS trial in Iceland (photo credit: Bertrand Bouchard).

The research boats used in Iceland and Antarctica were sailing vessels (12 m long, 1.7 m draft, 61 horse power engine and 20.1 m long, 2.2 m draft, 215 horse power engine, respectively) while in Madagascar we used a fibreglass boat (6.3 m long, 0.3 m draft and 140 horse power engine). At the beginning of the trial, we deployed the test solution from the boat at low speed (3–5 knots) in order to create a stimulus line of about 30 m in length. During the deployment, we released a recording platform in the middle of this line in order to mark the centre of the stimulus zone, since the red colour progressively disappeared within 10–15 minutes as the powder sank and dispersed in the water. The recording platform consisted of a floating Styrofoam platform (120 x 60 x 5 cm) equipped with an omnidirectional hydrophone (model C57, Cetacean Research Technology, Seattle, USA) connected to a digital audio recorder (acquisition at 96 KHz 24 bits on a Zoom H1 recorder, Zoom Corporation, Tokyo, Japan) and two underwater cameras (GoPro Hero 4, GoPro Inc., San Mateo, USA) facing forward and backward with an angle of 30° downward (Fig 2). This recording platform allowed the analysis of the whales’ vocalizations as well as their underwater behaviours in the vicinity of the stimulus area.

#### Experiment D: DMS solution

We prepared a 0.2 M DMS solution each day by diluting pure DMS (Purity ≥ 99%, Sigma-Aldrich, Munich, Germany) in commercial sunflower oil. This approach slowed the evaporation of DMS, a highly volatile compound (vapour pressure of 53.7 kPa at 20°C). While this concentration of DMS is much higher than that found in surface waters (where DMS rarely peaks over 20 nM during summer months [35,36]), we used it because this concentration has been previously shown to attract seabirds [5,37]. The control solution only consisted of commercial sunflower oil.

We poured the test and control solutions inside a floating round diffuser made of stainless steel-reinforced orange Styrofoam tubes with a diameter of 6 cm. A 30 cm wide polyvinyl chloride membrane was hung below the tubes in order to retain some of the oily solutions within the diffuser during the trial, and a 1 kg weight tied 40 cm under the device by three polyester straps worked as an anchor, preventing wind-drift (Fig 2). This floating diffuser limited spatial dispersion of the solution and created a focal point of highly-concentrated stimulus that was used by the observers as a visual reference. The same recording platform previously described was attached to the diffuser by a 1 m strap.

### Behavioural observations

**Fig 3:**
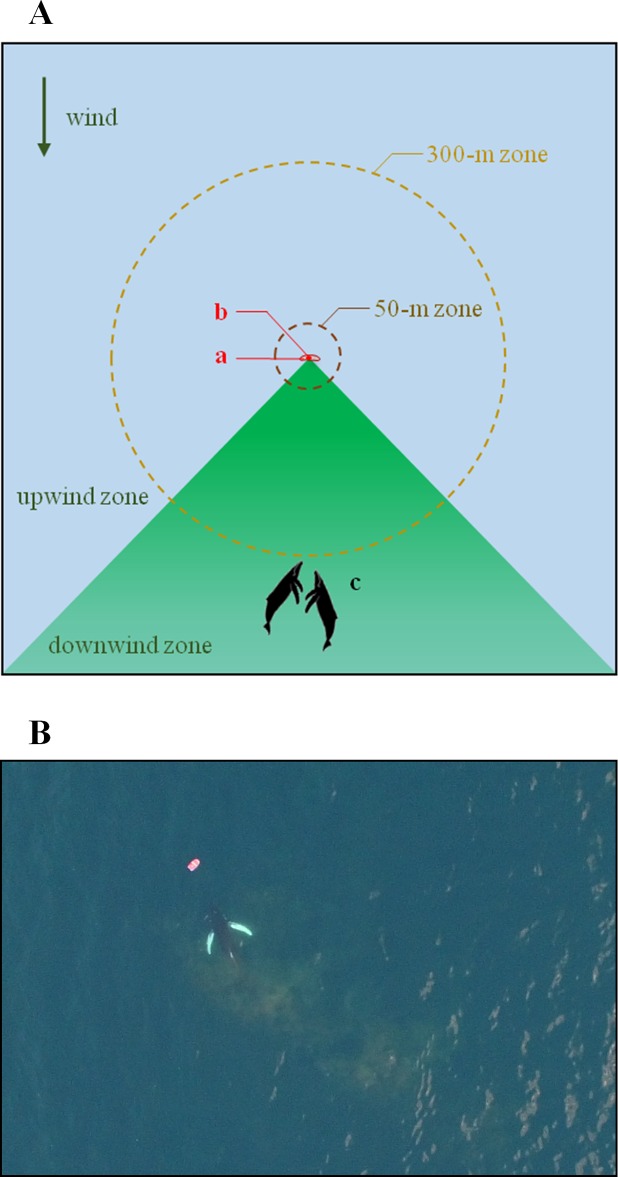
Illustration of the chemical exposure protocol. (**A**) Schematic illustration of the initial conditions for each exposure trial. For experiment K, the stimulus (**a**) was released along a 30 m line, with the recording platform (**b**) (equipped with a hydrophone and two underwater cameras) in its centre, approximately 300 m from the closest group of whales (**c**). The same protocol was used for experiment D, except that the stimulus was poured into a chemical diffuser (see Fig 2) attached to the recording platform by a 1 m long strap. Two exposure zones were considered in the behavioural analyses: a close exposure zone with a 50 m radius (dark red) and a medium exposure zone with a 300 m radius (orange). The whales’ dimensions are not to scale. (**B)** Drone photograph of a humpback whale swimming through a line of krill extract toward the recording platform (photo credit: Bertrand Bouchard).

The research boat was placed approximately 300 m upwind (i.e. into the wind) from the closest targeted whale(s) in order to maximize the exposure of the animals to the chemicals (Fig 3). The chemical stimulus, or its respective control, was then released in the water in randomized order. Once the experimenter deployed the stimulus at the back of the boat, out of sight of the observers (who were therefore blind to the treatment), the boat navigated to a distance about 200–300 m away from the stimulus zone and the captain stopped the engine. The two cetacean observers then started to record the position and behaviour of each whale or group of whales every minute for 22 minutes (determined by the operating time (i.e., battery life) of the aerial drone used to observe and film each trial, as described below) using 7x50 binoculars. We defined the position of a whale group as the centroid of the positions of all its members. As the chemical diffuser was placed 300 m upwind from the closest group, we also recorded the initial distance to the stimulus from all the other targeted groups. We also recorded the appearance of new, non-targeted groups that were not observed at the beginning of the trial.

We defined two zones around the stimulus area: a close exposure zone with a 50 m radius, and a medium exposure zone with a 300 m radius. When a group crossed these limits, we recorded the time spent since the beginning of the trial in order to measure the duration of its presence within each zone. We also counted the respirations (blows) from each whale or group of whales, as well as surface behaviours indicative of an exploration of the area. The surface behaviours we considered were: stopping (a marked decrease in navigation speed) and diving under the stimulus (a short dive in the close exposure), as well as non-vocal communication behaviours including breaching (leaping completely or partially out of the water), head-, pectoral- or tail-slapping (slamming the head or fin down on the water) [38,39].

For the duration of each trial, one of us (BB; an experienced drone pilot) flew an unmanned aerial vehicle (or drone) equipped with a 2.7K HD video camera (Phantom 3 advanced, DJI, Shenzhen, China) above the stimulus area. This allowed detailed information on the whales’ behaviour to be communicated to the observers in order to assist them in their data collection, as aerial observations can significantly improve the observational capacity in cetacean research compared to boat observations [40]. It also provided the observers with accurate data on the whales distance to the stimulus by estimating the centroid position. The position of the recording platform/diffuser was set as the drone homepoint and was updated every 3 minutes to correct for drifting. The minimum flight altitude was set to 50 m in order to ensure it was not seen or heard by the whales [41].

Birds can affect whale foraging behaviour [42], and therefore a trained ornithologist equipped with 7x50 binoculars recorded the presence and behaviour of any birds around the stimulus area. For each group of birds, the species and number of individuals were recorded, as well as any specific flight pattern including circling (the bird circles over the area), zigzagging (one or more turns of > 45°), flying down (the bird loses altitude, usually suddenly) and landing on water [43].

At the end of each 22-minute trial, the experimenter recorded the trial conditions including the stimulus type (krill extract, DMS, or their respective control), the GPS position and the environmental conditions (wind force, swell height, precipitation and visibility). The floating devices were then retrieved, and the boat was moved to another zone upwind in order to run a new trial on a different group of whales. The next trial was started after a period of at least 1 hour and in an area at least 2 km upwind from the previous trial, in order to avoid any potential disturbance from the stimulus used in the previous experiment and to avoid including the same group of whale in successive trials. In total, we ran 56 trials in humpback whale breeding (26 trials) and feeding grounds (22 trials in Iceland and 8 in Antarctica) during the four field surveys (Table 1). We could unfortunately not perform D experiments in Antarctica due to logistical and permit issues.

**Table 1:** Dates and number of trials for each type of chemical exposure experiment in the three study sites.

Study site	Dates	Experiment K		Experiment D	
		Krill	Control	DMS	Control
**Iceland**	14–30 August 2016	6	3	6	7
**Antarctica**	24 December 2016–01 January 2017	5	3	*Not tested*	
**Madagascar**	24–28 June 2015 & 30 June– 15 July 2016	9	5	7	5

### Vocalizations tracking and features extraction

We investigated the effects of exposure to prey-related chemicals on whale acoustic activity with an automated cluster-based detector. This method allowed the sounds produced by the whales to be screened quickly, and permitted all vocalizations from a recording to be described quantitatively, dated, and clustered. The detector binarized each pixel of the time-frequency spectrogram of the first 10 minutes of the recording by filtering pixels with higher frequencies than background noise; this method has the additional advantage of excluding incomplete and low-amplitude vocalizations emitted by whales located far away from the stimulus area. A second process filtered pixels forming continuous time-frequency tracks which are characteristic of animal vocalizations. The tracks were then verified by visual and auditory inspection. Fifteen acoustic descriptors were extracted from each vocalization (minimum, maximum, median and mean frequency, vocalization duration, minimum, maximum, median and mean duration of velocity, minimum, maximum, median and mean duration of acceleration). We projected these 15 variables into clusters within a lower dimension space using a t-SNE (Distributed Stochastic Neighbour) algorithm in 2 dimensions. This method is better suited to reduce the dimensionality of acoustic signals than principal component analysis because it does not assume linear relationships among the 15 descriptors [44]. We used each vocalization’s coordinates in the t-SNE space to build Bayesian Non-Parametric clusters (BNP). This clustering method has been found to be optimal for cetacean bioacoustics analyses [45], allowing the classification of vocalizations with a high degree of intraclass similarity (resemblance within a cluster) and a low degree of interclass similarity (dissemblance between clusters).

We measured the quality of the clusters with Normalized Mutual Information scores (NMI) [46,47] in order to quantify the correspondence (i.e. mutual dependence or mutual information) between acoustic and chemical variables. The NMI score is calculated as NMI (X,Y) = $\frac{I(X,Y)}{\sqrt{H(X)H(Y)}}$, where X is the cluster of the projected acoustic patterns (BNP: data clustering into disjoint subsets), Y the stimulus condition, H the entropy (i.e. measure of the amount of information [47]) and I the mutual information. The NMI ranges from 0 (the two variables do not share any information) to 1 (one-to-one correspondence between acoustic clusters and stimulus condition).

### Data analysis

All analyses were performed in R [48]. A summary on data collection and analysis can be found in Table 2.

**Table 2:** Summary of data collection and analysis for the behavioural response experiments.

Data collection	Measured data	Data analysis
Visual observations from the boat using binoculars, assisted by UAV	Number of non-targeted whales that appeared during the trial	GLM (Poisson distribution)
	Time spent by the whales in close exposure zone (50 m radius)	Tobit models for zero-inflated data
	Time spent by the whales in medium exposure zone (300 m radius)	Tobit models for zero-inflated data
	Number of whale blows	Linear model
	Whale surface behaviours	Two-tailed Fisher’s exact test
	Bird counts	GLM (negative binomial)
	Bird flight behaviours	Two-tailed Fishers’ exact test
Underwater cameras	Whale underwater behaviours	Descriptive analysis
Hydrophone	Whale acoustic data	Automatic spectral tracker and clustering

As explained above, we took a whale group as a single sampling unit for all statistical analyses. First, we tested whether the chemical stimulation could have attracted whales that were not in sight during the pre-trial observation. The number of non-targeted whale groups that appeared per trial was analysed using a generalized linear model (GLM) with a Poisson distribution including the stimulus condition and several control variables such as the study area, wind speed and swell, as well as the presence of birds, their numbers and flight behaviours.

We also examined the influence of krill extract and DMS on several response variables. First, we considered the time spent by the whales in each of the two zones (50 and 300 m zones) assumed to reflect their exploration of the stimulus area. This variable had a zero-inflated Gaussian shape because several whales groups did not enter any of the zones. This issue was overcome by testing stimuli effects in a Tobit regression (censReg R package) [49]. One global model was first created for all trials. We added study area, distance at start, time of arrival, wind speed, swell, group size as well as bird counts and their flight behaviour as control variables. In order to compare the whales’ exploration of the stimulus zone in the three different study areas, we also used a Tobit regression for each of the sites. We then tested the influence of the food-related stimuli on the whales’ respiratory rates (defined as blow counts within a group divided by the product of the group size and the time the group was observed during that surface series) using a linear model on square root-transformed values. For each of these models, we created a maximum model with all independent variables and used the *dredge* function (MuMIn R package) [50] to test which variable combinations resulted in the most parsimonious model based on the Akaike information criterion (AIC), considering a model to be substantially better than another if its AIC was lower by at least 2 AIC units [51]. For both experiments (K and D), the effect of the stimulus condition was compared to its control by post-hoc multiple comparison using the *glht* function (multcomp R package) [52].

Finally, we tested the influence of the chemical stimulation on the whales’ surface behaviour. We built a series of six 2 x 2 contingency tables for each experiment (K and D), with one variable being stimulus or control, and the other being the number of groups that displayed, or did not display, each of the six surface behaviours (stopping, diving, breaching, and head-, pectoral- or tail-slapping). The differences between proportions were tested using a two-tailed Fisher’s exact test.

As a side-analysis, we investigated whether the birds observed in the stimulation zone responded to the chemical stimulation and/or the whales’ behaviour. The effect on bird counts was evaluated using negative binomial regression using the *glm*.*nb* function (MASS package) [53] because of over-dispersion of the data and using the same model selection method as for whales’ behaviour, including control variables such as wind force, study area, whale counts and surface behaviours, as well as time of day. The influence of the chemicals on the birds’ behaviour pattern was calculated using the occurrence of each flight pattern during a trial. This allowed us to avoid any bias from a potential cumulative effect due to local enhancement in birds (i.e. the use of congeners as distant visual cues while foraging), a well-described behaviour in seabirds [54,55]. This variable followed a binomial distribution as it was counted as absent or present (0 or 1) during each trial. These data were pooled and organized in 2 x 2 contingency tables incorporating the stimulus conditions and the differences between proportions were tested using a two-tailed Fisher exact test.

## Results

### Number of whale groups

**Fig 4:**
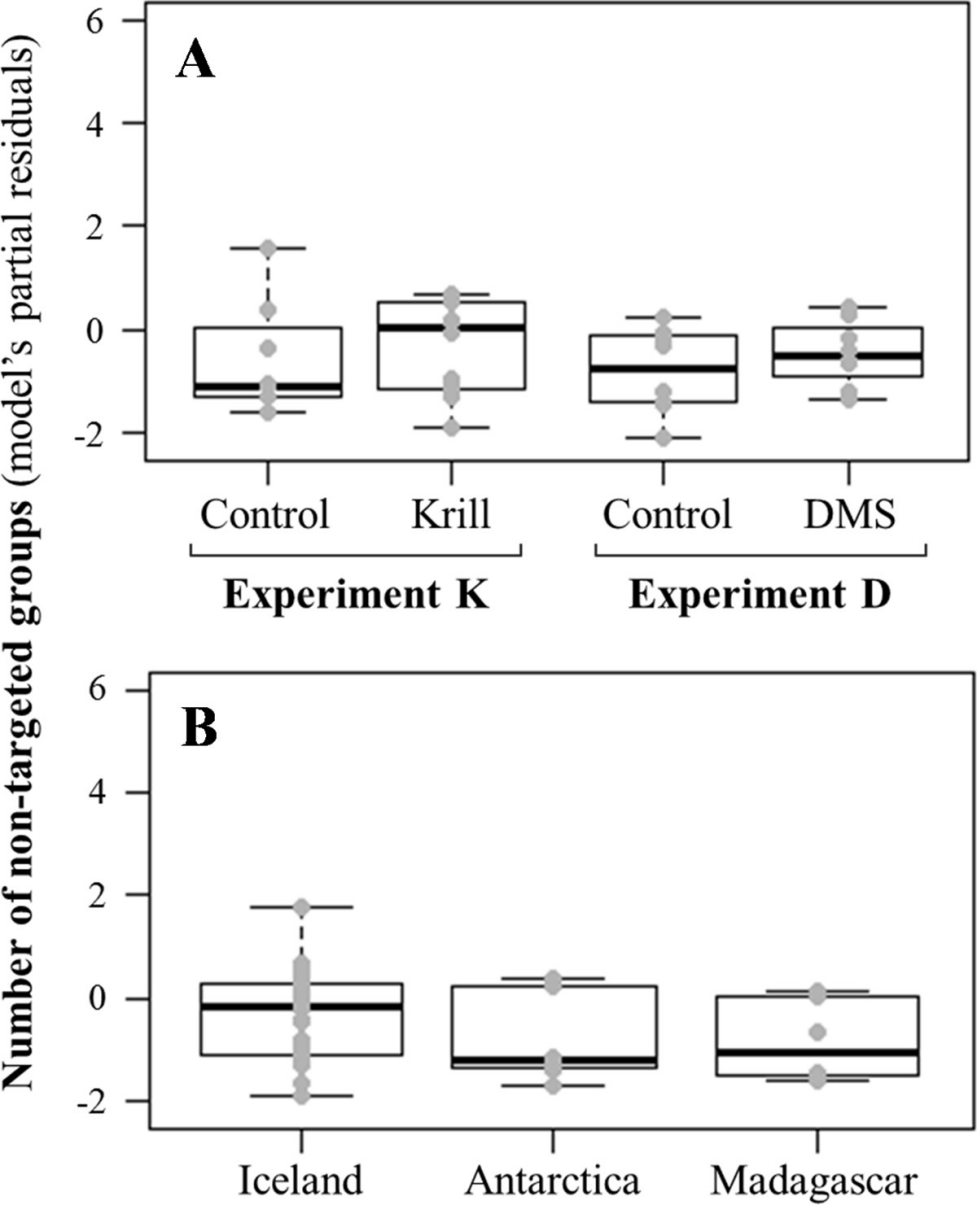
Partial regression plots showing the relationship between the number of new, non-targeted whale groups (i.e. not observed at the beginning of the trial) and main experimental variables. There was no significant effect of the exposure experiment or the stimulus condition on the response variable (**A**). The effects of control variables such as the study area (**B**), the time of day, wind force, swell and bird counts or behaviours, were also found to be non-significant.

A total of 113 humpback whale groups were included in our analyses (range: 1–5 per trial), totalling 164 individuals (range: 1 to 4 individuals per group, mean ± S.D.: 1.45 ± 0.45). Only one other whale species (a minke whale, *Balaenoptera acutorostrata*) was observed in the stimulus zone during one krill trial in Antarctica, and this was excluded from the analysis. Across all our trials, between 0 to 3 new whale groups (average: 0.77 ± 0.87) that were not targeted at the beginning of a trial (because they were not detected during the pre-trial observation phase) appeared in the area, potentially attracted from a long distance. However, our GLM showed that this number of non-target study groups was either not significantly affected by the stimulus condition (estimate = 0.188, 95% CI = -1.630 − 2.008, p = 0.993 for krill extract and estimate = 0.242, 95% CI = -1.112 − 1.598, p = 0.967 for DMS) or by the control variables (study area, time of day, wind force, swell, bird counts and flight behaviours) (Fig 4).

### Time spent in the stimulus zone

#### Global model

**Fig 5:**
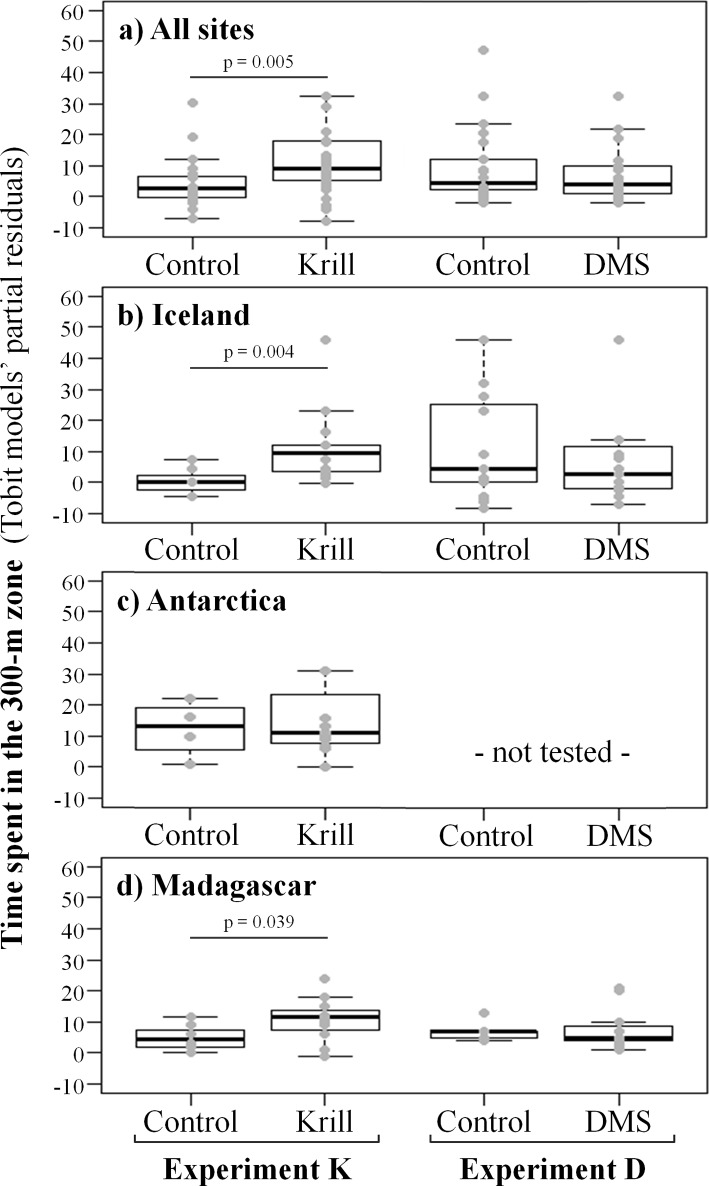
Partial regression plots showing the relationship between the time spent by humpback whales in the 300 m zone around the recording platform/chemical diffuser and the type of chemical stimulation. A significant increase compared to control was observed during K (krill) experiments in Iceland (**B**) and Madagascar (**C**) as well as when all sites were considered (**A**), but not in Antarctica (**D**). No significant effect of DMS was observed in any of the study sites.

We considered the time spent by the whales within the two exposure zones around the recording platform/diffuser (50 m and 300 m zones) as indicative of their exploration of the chemical source. The most parsimonious Tobit model for the 300m zone included the chemical stimulus and three control variables: study area, initial distance and time of day (McFadden pseudo R-squared = 0.14). The stimulus effect was highly significant in experiment K (Fig 5): during krill extract trials, whales spent about 8 minutes more near the stimulus than during control trials (estimate = 8.01, 95% CI = 1.84–14.18, p = 0.0048). However, the same was not true for DMS (estimate = 0.76, 95% CI = -5.97–7.49, p = 0.99). The study area had no significant effect on the time whales spent in the vicinity of the diffuser. As expected, the model found a highly significant negative effect of the initial distance on this response variable (estimate = -0.029, 95% CI = -0.0041 –-0.017, p < 0.001). The time of day also had a significant negative effect, the whales spending less time in the 300 m zone in the afternoon than in the morning (estimate = -15.14, 95% CI = -25.70 –-4.57, p = 0.003). The influence of the chemical stimulus on the time spent in the 50m zone followed the same trend, except that the positive effect of krill extract was not significant (estimate = 4.18, 95% CI = -1.08–9.46, p = 0.17).

#### Intersite differences

As explained in the introduction, the three study areas differed in terms of the whales’ reproductive state (breeding vs. feeding grounds) and in terms of prey availability (no prey in Madagascar, krill and fish in Iceland, and exclusively krill in Antarctica). We tested whether these differences could affect the whales’ response to the two food-related chemicals. We observed a response of higher intensity in Iceland than in Madagascar (estimate = 13.92, 95% CI = 3.28–24.56, p = 0.0042 and estimate = 8.03, 95% CI = 0.25–15.81, p = 0.039, respectively) and no effect was found in Antarctica (estimate = -5.56, 95% CI = -14.08–2.95, p = 0.20) (Fig 5). In the 50 m zone, a marginally non-significant effect was only found in Iceland (estimate = 7.57, 95% CI = -0.49–15.65, p = 0.073). Exposure to DMS did not induce any response in the two study areas where it was tested (Iceland and Madagascar).

### Respiratory rate

**Fig 6:**
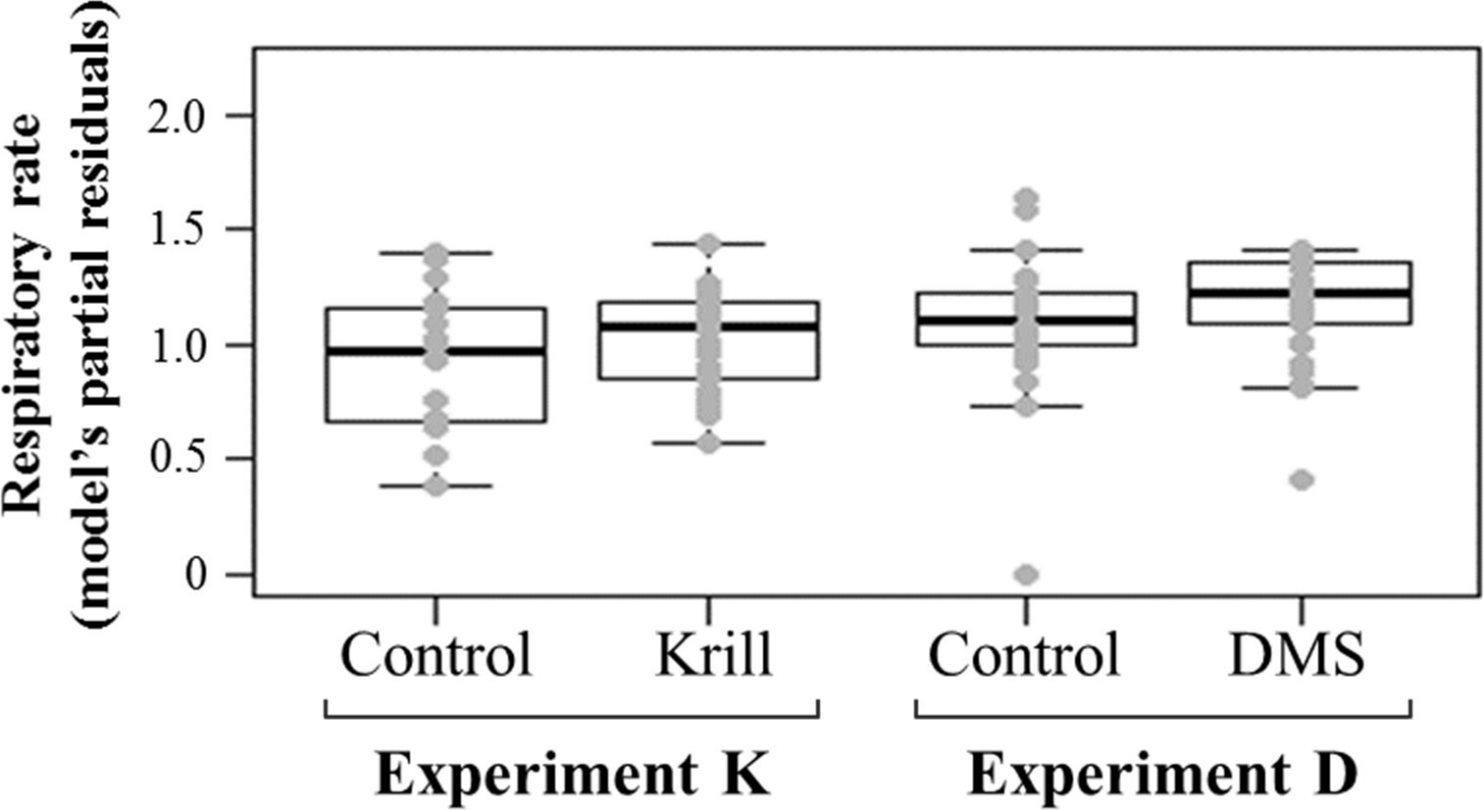
Partial regression plot showing the relationship between the humpback whales’ respiratory rate and the type of chemical stimulation. No significant difference was found between krill extract or DMS trials and their respective controls (p > 0.05).

We calculated the average respiratory rate of the whales in each group in order to test whether respiratory rate changed in response to exposure to airborne food-related chemicals. The most parsimonious linear model for respiratory rate included two explanatory variables: chemical stimulus and study area (R-squared = 0.50). Chemical stimuli did not significantly affect the response variable compared to their respective controls (estimate = 0.078, 95% CI = -0.114–0.269, p = 0.71 for krill extract and estimate = 0.056, 95% CI = -0.131–0.245, p = 0.85 for DMS) (Fig 6). The study area had a strong influence on the respiratory rates, being higher in Iceland than in both Antarctica and Madagascar (estimate = 0.384, 95% CI = 0.185–0.582, p < 0.001 and estimate = 0.518, 95% CI = 0.649–0.387, p < 0.001, respectively).

### Surface and underwater behaviours

**Fig 7:**
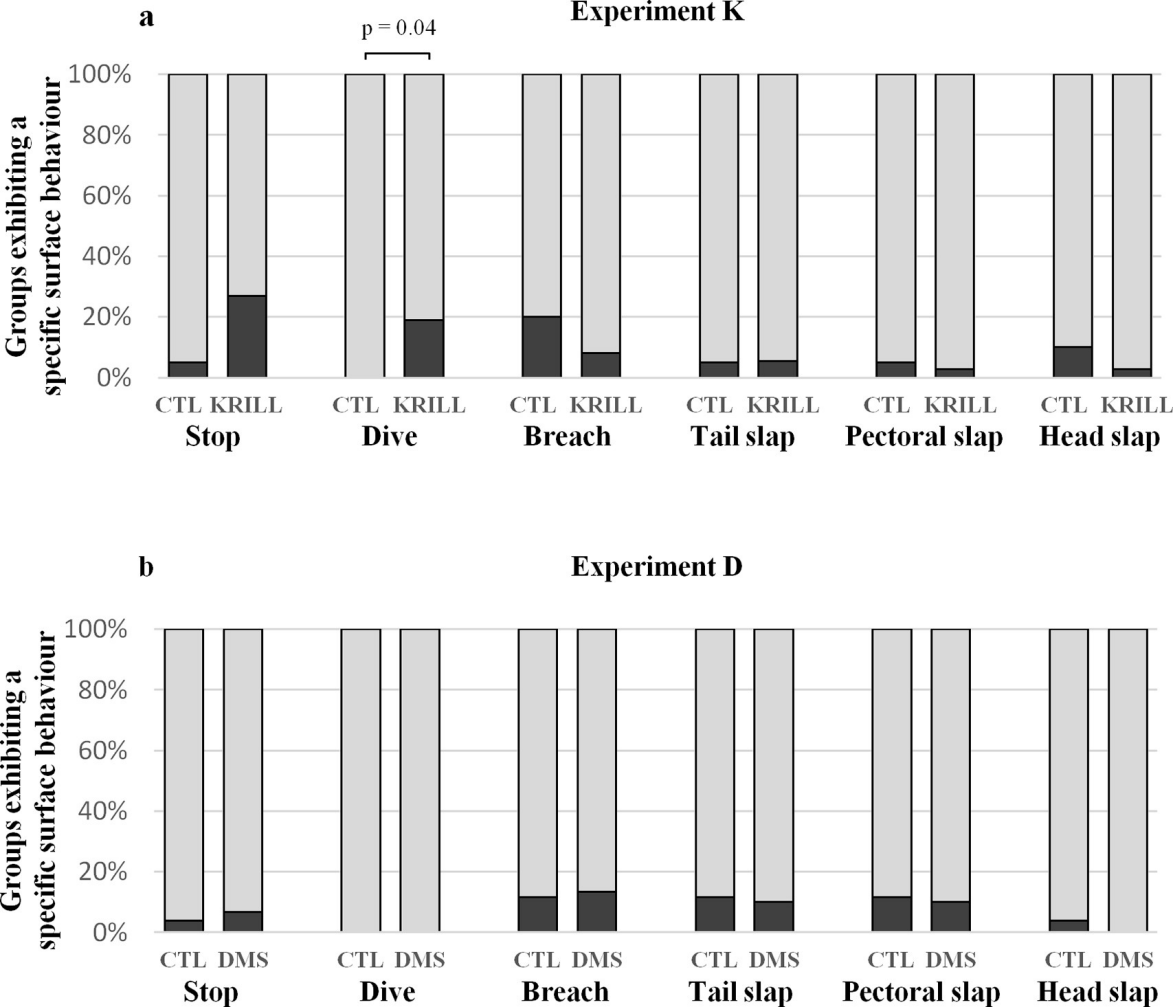
Occurrence of specific surface behaviours in humpback whales exposed to a chemical stimulation or a control during behavioural response experiments. (**A**) Krill extract versus control (CTL) including 20 and 37 groups, respectively. (**B**) DMS versus control (CTL) including 26 and 30 groups, respectively. Diving under the stimulus was the only behaviour significantly affected during chemical stimulation with krill extract when compared to control trials (occurrence in 18.9% vs. 0.0% of whale groups, respectively, n = 57, p = 0.04, two-sided Fisher’s exact test).

We counted all surface behaviours since they could potentially be affected by the whales’ detection of the food-related chemical stimulus (Fig 7). In experiment K (Fig 7A), we observed that the whales exposed to krill extract dived under the recording platform/diffuser significantly more often than those exposed to the control solution (18.9% vs. 0.0%, n = 57, p = 0.04, respectively, two-sided Fisher’s exact test). There was also a marginal increase in the frequency of navigation stop near the stimulus during krill extract trials (27.1% vs. 5.0%, n = 57, p = 0.08). Non-vocal communication behaviours were not affected by the exposure to krill extract. In experiment D, there was no significant difference in the occurrence of any behaviour between DMS and control trials (Fig 7B).

In all three study areas, underwater visibility was less than 10 m due to turbidity. Therefore, although 26 whale groups entered the close exposure zone (within 50 m around the recording platform/diffuser) in total, only two of them (3 individuals in total) were captured by the recording platform’s cameras during two krill extract trials in Skjálfandi Bay, Iceland. One individual approached underwater and surfaced 7 m from the diffuser. It then swam under the surface back and forth within 20 m around the floater for 55 sec, breathing twice, before swimming away. The two other individuals formed a unique group. After they approached and breathed 5 m from the floater, they slowly swam down to a depth of approximately 10 m during 12 seconds, before coming back to the surface to breathe again. No mouth opening or other specific behavioural displays were observed underwater for any of these individuals.

### Vocalizations

Our algorithm detected a total of 2314 vocalizations from the acoustic recordings, all of them in Madagascar (no vocalizations were detected in Iceland nor in Antarctica). In our analysis we considered the 1000 longest (i.e. most complete) calls, as these were emitted by the closest whales to the stimulus diffuser where the hydrophone was attached (sound reception and power being negatively correlated to the distance to the source). The clustering with maximum NMI (NMI = 0.40) was obtained with two BNP clusters. The first cluster (named ‘0’) is dominated by sounds produced in both control conditions, while most of the calls in the second cluster (named ‘1’) were produced during exposure to the two food-related chemical cues (krill extract or DMS) (S1 Fig). These results suggest that the vocalizations produced during exposures to chemical cues have different acoustic features than the ones produced during control trials.

### Influence of the birds as visual cues

Of the 471 bird groups counted during the 56 trials, 399 (83.7%) were northern fulmar *Flumarus glacialis* sighted in Iceland. The remaining 28 groups encompassed 14 species, differing in each area (S1 Table). The general linear model showed that the bird count per trial was not significantly affected by the chemical stimulation, but only by the study area (Iceland > Antarctica > Madagascar, p < 0.001) and by the occurrence of whales’ breaching (estimate = 1.897, 95% CI = 0.661 − 3.133, p = 0.002) (S2A–S2D Fig). Chemical stimulation had no significant effect on the birds’ flight pattern except a marginally non-significant positive effect of krill extract on the occurrence of zigzag flight (p = 0.06, two-sided Fisher’s exact test on contingency table) (S2E and S2F Fig).

## Discussion

In this study, we investigated the behavioural and acoustic responses of humpback whales exposed to two food-related chemicals, krill extract and DMS, released into the water. We found that humpback whales reacted to these chemicals, providing the first experimental evidence for a functional chemosensory system in this species. Of the two different chemical stimuli we used, the whales’ reactions towards krill extract were much stronger than those shown towards DMS.

Exposure to krill extract resulted in whales spending a significantly longer time in the stimulus area, when compared to the controls. Moreover, the whales dove more frequently and tended to stop their navigation more often in the vicinity of the chemical stimulus during krill extract trials than control trials. These results provide strong evidence that the animals detected the stimulus and approached its source, presumably in search for the related prey. Attraction to krill-derived chemicals would increase their probability of finding krill aggregations, which are patchily and unpredictably distributed [56,57]. The increased rates of stopping and diving behaviour in relation to the krill extract suggest that the whales displayed a greater degree of exploratory behaviours after they had detected the chemical stimulus. After a chemical cue has been detected, exploring the area at low speed may enable them to better perceive other prey-related cues at short distance using other senses such as vision, mechanoreception and audition [1,58]. This corroborates other behavioural studies that have shown that humpback whales, like other baleen whales, swim at a relatively low forward speed during foraging, only accelerating during underwater lunges [59,60]. The detection of chemical cues could enable them to maintain proximity to the highest densities of their prey [26,61], a behaviour which appears crucial to maximize their energy intake during summer months before migrating towards their breeding grounds where there is no food available.

Response to krill extract compared to control trials varied among the three study areas. While the difference in the time spent in the stimulus area was the highest in Iceland, no difference was found in Antarctica. One possible explanation for this contrast may be that the krill density in the western Antarctic Peninsula is usually several orders of magnitude higher than in Northern Iceland (62,000 vs. 8.8 g per 1000 m^3^) [62,63]. Therefore, the high levels of krill-derived chemicals already present in the Antarctic environment may have hidden the signal created by the experimental stimulus, which represents a relatively small quantity (equivalent of approximately 20 kg of fresh krill) compared to what is found naturally in the surrounding waters. In Madagascar, the observed increase in the whales’ exploration time, even if it was less marked than in Iceland, was not expected because their behavioural and physiological state is primarily oriented towards breeding at this time of the year. This species has however been reported to feed at low latitudes during migration [64], so this reaction may suggest that the detection of a prey-related cue could trigger opportunistic foraging, even in areas where feeding usually does not occur. Our data also show that the attraction towards krill extract progressively decreases from early morning until afternoon, which could be due to a circadian cycle in the whales’ feeding activity. Indeed, diel changes in feeding behaviour (linked to the behaviour and distribution of prey, in particular to their vertical migration during the day and the night) have previously been documented in humpback whales in both Antarctic and North Atlantic waters [32,62].

Krill extract is a mixture of various individual chemicals that could be detected by the whales’ chemoreceptors. A chemical analysis carried out on homogenized Antarctic krill samples showed that it contains several classes of volatile compounds that could be ligands for the whales’ olfactory receptors, including esters, aldehydes, ketones, pyrazines, hydrocarbons as well as DMS [65]. We also expect it to include a significant amount of peptides and amino acids since the krill used in our experiments is an enzymatic (subtilisin EC 3.4.21.14) hydrolysate. Interestingly, prey metabolites and dissolved free amino acids have been described as chemical cues increasing feeding activity in both bony fish and sharks [3,66,67].

While it has been speculated that baleen whales use DMS as an indicator of prey aggregation [13,16], we did not observe any differences in the whales’ exploration of the stimulus area, or their surface behaviour, between the DMS and control trials. One possible cause could be the concentration of DMS used in this experiment, which was much higher than what is found in the marine environment, making the stimulus too strong to be recognized as a natural foraging cue by the whales. However, similar concentrations trigger foraging behaviour in several species of marine birds [5,37]. Also, humpback whales are foraging generalists and feed on a wide range of prey [18], some of which do not consume DMS-producing phytoplankton. They may thus be less sensitive to this stimulus than other mysticete species that feed exclusively on DMS-producing phytoplankton grazers. For example, blue (*Balaenoptera musculus*) or bowhead whales are krill specialists targeting mostly euphasiids (which have been shown to increase DMS production through their grazing activity [68]). We therefore predict that these species would be more likely to exhibit behavioural responses when exposed to DMS. Another plausible explanation would be that in comparison to krill for example, DMS is used for navigation over a much larger scale (ranging from tens to hundreds of kilometres), being associated with predictable oceanic features such as seamounts, shelf breaks or upwelling zones [69]. This molecule may thus be less useful for whales when it comes to identifying the specific location of an odour source at a finer scale (hundreds of metres), explaining why the whales investigated in this study did not express behavioural reactions [1]. Further research is needed to study the potential use of DMS as a navigational cue over long distances in humpback whales. This would require the combination of GPS tracking data with particle dispersal models and DMS production models, a method which recently allowed to better understand the importance of olfaction in migratory birds [70,71].

Our acoustic analyses were based on an automatic method that only detected calls at the study site in Madagascar. These results confirm the strong difference in the whales’ acoustic activity between breeding grounds, such as Madagascar where they produce a high quantity of songs, and feeding grounds such as those around Iceland and the Antarctic Peninsula, where far fewer calls are emitted [18]. The results also suggest that the whales’ vocalizations were modified in Madagascar when they were exposed to krill extract or DMS. Modification of humpback whales’ acoustic activity in their breeding areas is known to occur in response to anthropogenic noises [72–74]. Similarly, detecting a food-related chemical in an environment where prey is not usually encountered may represent an unusual stimulus for the whales and could trigger a change in their acoustic activity.

Our experimental design did not allow us to determinate whether DMS or krill extract were perceived in air, in water or both. Over longer distances (hundreds of metres from the diffuser), the whales were exposed mostly to volatile compounds because molecules travel much faster in air than in water, and so could have detected these molecules using their olfactory system (see Introduction). Most mammals change their respiratory pattern following an olfactory stimulus, optimizing odorant sampling by sniffing [75]. The absence of a significant difference in respiratory rates between stimulus and control trials may reveal that whales, like birds, lack sniffing-associated behaviours, or that they use subtle air sampling behaviours that differ from blows and that our observers could not detect from a distance. Whales that approached within tens of metres of the recording platform/diffuser would also have been exposed to soluble compounds in the water. While the three whales captured by the underwater cameras did not exhibit obvious open-mouth behaviour or other foraging patterns, groups that swam in the vicinity of the diffuser could have perceived chemical stimuli in water, presuming that they possess taste receptors in their oral cavity or elsewhere on their bodies. Although data on the gustatory capabilities of mysticetes are still scarce, a recent study identified fungiform papillae on the tongue in a gray whale (*Eschrichtius robustus*) which have very similar characteristics to those involved in taste perception in terrestrial mammals [19]. Furthermore, functional (i.e. non-pseudogenized) taste receptor genes have been identified in mysticete and odontocete cetaceans [76], and behavioural experiments have revealed that odontocetes are able to react to various taste stimuli [77–80]. We therefore think it is likely that mysticetes also possess a functional gustatory system. More work would however be required and this will be an important area for future investigation.

Taken together, our results strongly suggest that humpback whales, and probably other mysticete species, partly rely on chemoreception to localize their food, as it has been shown in other marine predators such as sharks, bony fish, sea turtles, oceanic seabirds and seals [2,3,81,82]. They also corroborate the hypothesis that chemical cues, especially airborne molecules that can travel long distances in the windy marine environment, may be used for navigation in conjunction with other senses such as magnetoreception, audition, vision and somatosensory perception of oceanographic stimuli (e.g. temperature) [1]. Moreover, in some mysticetes including the bowhead and gray whales, the vibrissae located around the blowhole could improve the localization of the odour source, combining information from wind direction with chemoreception in the nasal cavity [83,84]. The perception of environmental volatile signals would be especially crucial for migratory mysticete species such as humpback whales that annually travel thousands of kilometres in the open ocean to reach localized feeding or breeding grounds. The importance of olfactory plumes originating from the migratory corridor for successful navigation has recently been shown in migratory gulls [70,71]. Our results also showed that while such birds are often associated with cetaceans at prey aggregations [42,85], their presence or behaviour did not affect the whales’ response. On the contrary, seabirds appeared in higher numbers when whales were breaching, providing further evidence of their use of cetaceans as visual cues to find feeding areas [86,87].

Finally, the present study gives new insights into baleen whale foraging ecology and provides behavioural evidence in support of their use of chemoreception in air and potentially in water. More experiments are now needed to assess the reactions of other mysticete species, especially krill specialists such as blue whales, to DMS or krill extract. Our findings also open up new opportunities for further research on the use of this sense in navigation at larger scales in cetaceans, as it has been recently achieved in migratory birds [70,71]. This new knowledge on mysticete chemosensory abilities could also find practical applications for cetacean management and conservation such as the use of chemical repellents in dangerous areas, including areas of high-density maritime traffic or fishing zones.

## Supporting information

S1 Table Bird groups observed during behavioural response experiments implemented in humpback whales’ breeding (Madagascar) and feeding grounds (Iceland and Antarctica). (TIF)

S1 Fig Analysis of the whales vocalizations emitted in Madagascar during exposure experiments. After dimensionality reduction (t-SNE) of all acoustic parameters, a Bayesian non-parametric clustering (BNP) was applied to the data. A maximum NMI score (0.27) was obtained using 3 clusters.
(TIF)

S2 Fig Birds’ presence and activity during exposure to food-related chemical cues. Using a generalized linear model with a negative binomial link, we found no difference in the bird count according to the type of chemical stimulation (**a**) or the number of individual whales in the stimulus area (**b**). However, the study area (**c**) and the whales’ surface activity (**d**) did have a significant effect on this response variable. Using two-sided Fisher’s exact test, no difference was found in the occurrence of specific flight behaviour in birds during experiment K (**e**) and D (**f**). ** p < 0.01, *** p < 0.001.
(TIF)
