# Supplementary figures and images for: Behavioural responses of humpback whales to food-related chemical stimuli
Source: PLoS One. 2019 Feb 26;14(2):e0212515. doi: 10.1371/journal.pone.0212515 (PMC6391047; doi:10.1371/journal.pone.0212515)

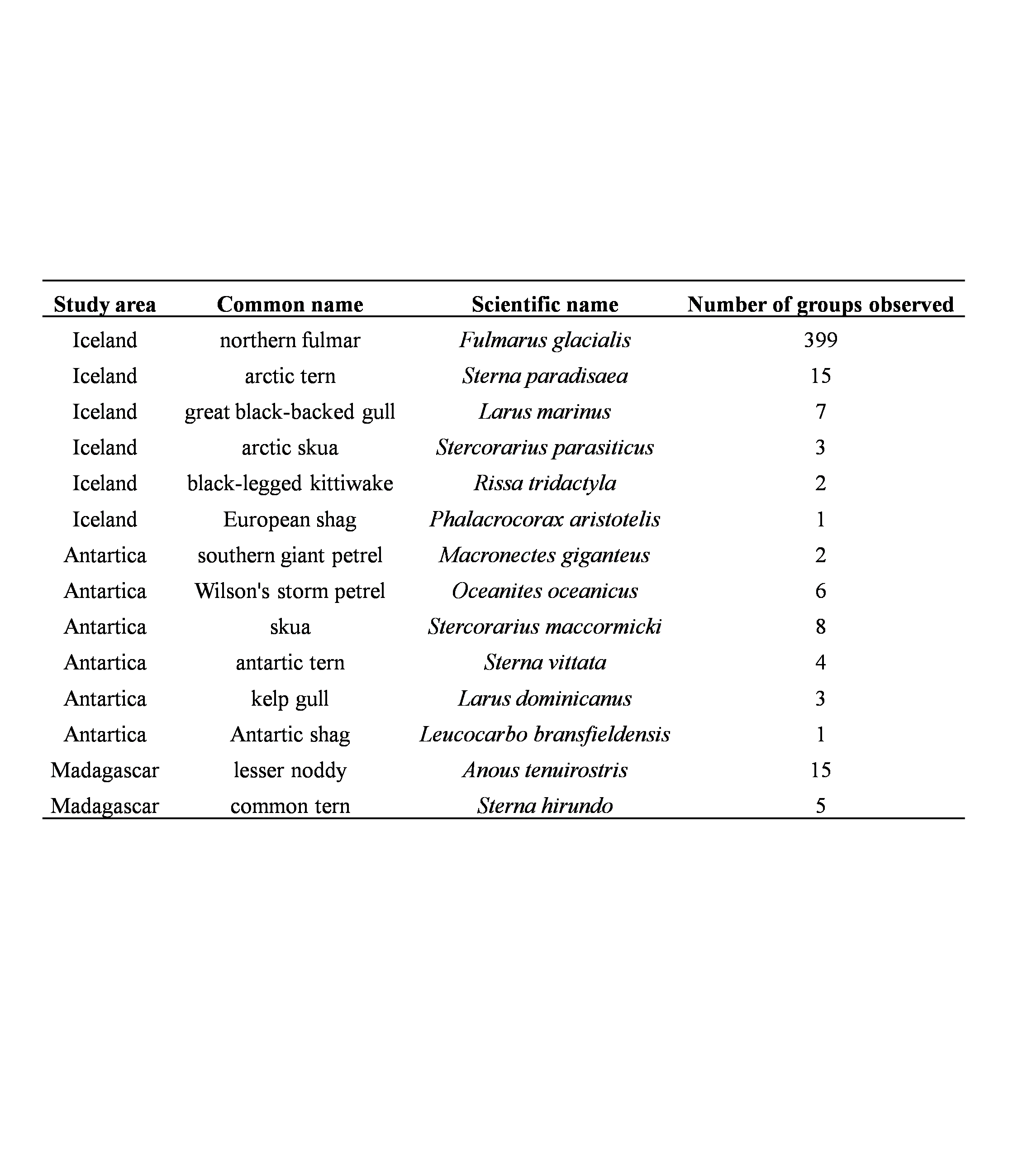

Supplement: S1 Table — (TIF) [file pone.0212515.s001.tif]

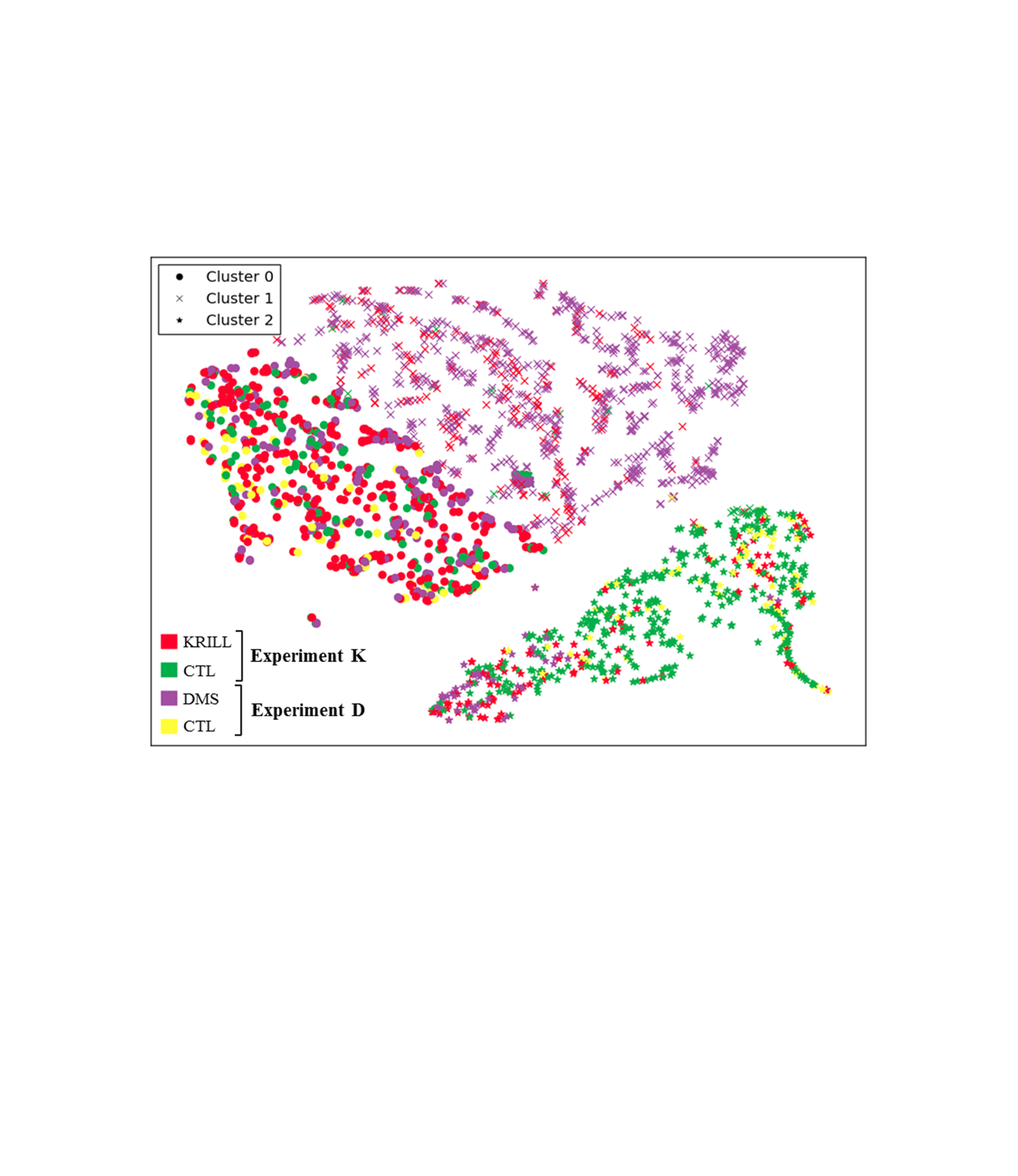

Supplement: S1 Fig — After dimensionality reduction (t-SNE) of all acoustic parameters, a Bayesian non-parametric clustering (BNP) was applied to the data. A maximum NMI score (0.27) was obtained using 3 clusters. (TIF) [file pone.0212515.s002.tif]

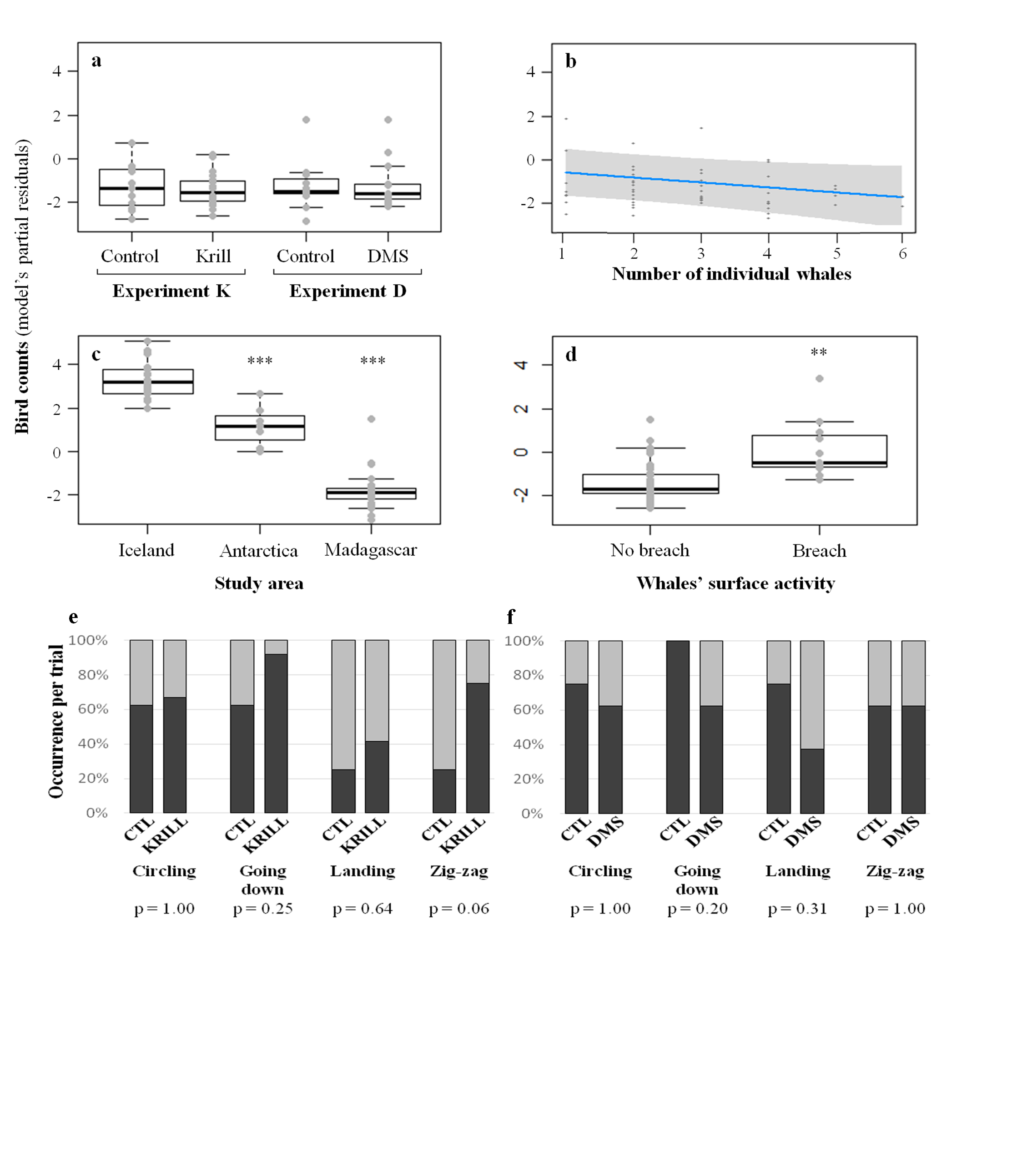

Supplement: S2 Fig — Using a generalized linear model with a negative binomial link, we found no difference in the bird count according to the type of chemical stimulation (a) or the number of individual whales in the stimulus area (b). However, the study area (c) and the whales’ surface activity (d) did have a significant effect on this response variable. Using two-sided Fisher’s exact test, no difference was found in the occurrence of specific flight behaviour in birds during experiment K (e) and D (f). ** p < 0.01, *** p < 0.001. (TIF) [file pone.0212515.s003.tif]
